# 489. Spatial transcriptomic analysis of placental tissue demonstrates differential gene expression in pregnancies with SARS-CoV-2 infection, SARS-CoV-2 placentitis, and controls

**DOI:** 10.1093/ofid/ofad500.558

**Published:** 2023-11-27

**Authors:** Leena B Mithal, Ramon Lorenzo-Redondo, Ashwin Sunderraj, Elisheva D Shanes, Jeffrey A Goldstein

**Affiliations:** Ann and Robert H. Lurie Children's Hospital of Chicago, Chicago, Illinois; Northwestern University, Chicago, Illinois; Northwestern University Feinberg School of Medicine, Chicago, IL; Northwestern University Feinberg School of Medicine, Chicago, IL; Northwestern University Feinberg School of Medicine, Chicago, IL

## Abstract

**Background:**

SARS-CoV-2 infection in pregnancy can cause placental abnormalities such as “SARS-CoV-2 placentitis” (histiocytic intervillositis, perivillous fibrin deposition, and villous trophoblast necrosis) that is irrespective of infection severity and associated with stillbirth. Mechanisms of SARS-CoV-2 placental injury are poorly understood. We investigated gene expression through next generation sequencing (NGS)-based spatial transcriptomics of placentas from SARS-CoV-2 infection and controls.

**Methods:**

Cases of SARS-CoV-2 in pregnancy with normal histopathology [n=4], pandemic timeframe controls with normal histopathology [n=2], and SARS-CoV-2 placentitis [n=3] were examined using Amsterdam Criteria and CD68 staining. RNA was extracted from FFPE tissue and underwent 10x Genomics Visium spatial NGS. Gene expression was analyzed while retaining tissue structure (Fig 1). Sequences and images were processed with Space Ranger 1.3.1 and R Seurat v.4.1.1.51. Raw counts were normalized using SCTransform. The Louvain algorithm and t-SNE algorithm were used for clustering and visualization. Differentially expressed genes were identified with Seurat and MAST. Various downstream pathway and cell type enrichment analyses were performed on transcriptional clusters.

**Figure 1.**
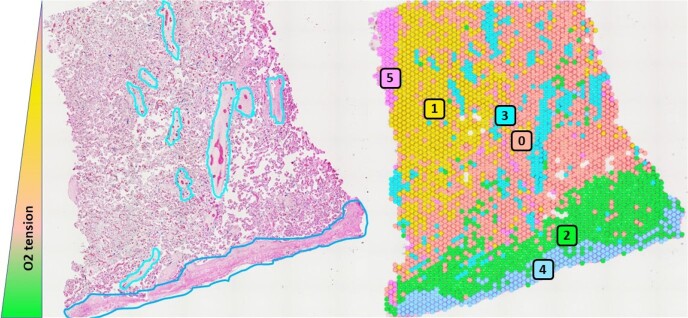
Anatomy underlies placental gene expression heterogeneity.

Section of a control placenta in H&E (left) and colored spots based on Louvain clustering of gene expression (right). The basal plate (dark blue, #4) is an area of fibrin and decidua adjacent to the uterine wall. Oxygenated maternal blood enters at the basal plate. Areas of terminal villi adjacent to the basal plate (green, #2) receive the highest oxygen with relative hypoxia further from the basal plate (salmon, #0 --> yellow, #1; pink, #5 is a subset of salmon). Stem villi (aqua, #3), which carry fetal blood to and from the placenta, form their own cluster.

**Results:**

Anatomic structures were related to gene expression clusters (basal plate, villi; Fig 1). Clinical data is in Table 1. Unique clusters were present in controls compared to SARS-CoV-2 placentas with normal histopathology (Fig 2). Genes upregulated in a distinct cluster in controls showed enrichment of macrophages and increased expression of cell regulation and viral responses, such as the interleukin 1 receptor-like 1 (IL1RL1), indicating dysregulation of normal immune responses in SARS-CoV-2 placentas. We found significantly upregulated genes in villous areas of placentitis (neutrophil degranulation and interferon signaling; Fig 3) compared to normal SARS-CoV-2 cases.

**Table 1.**
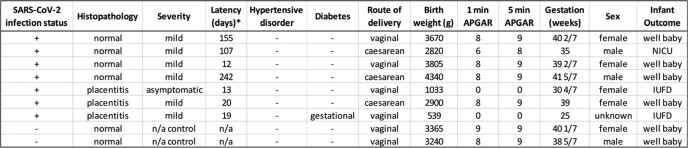
Clinical data and categorization of cases. * Latency = time between SARS-CoV-2 infection and delivery admission. Controls were defined as no SARS-CoV-2 infection in pregnancy, negative SARS-CoV-2 PCR on hospital admission screen, and negative anti-SARS-CoV-2 spike protein IgG/IgM at delivery.

**Figure 2.**
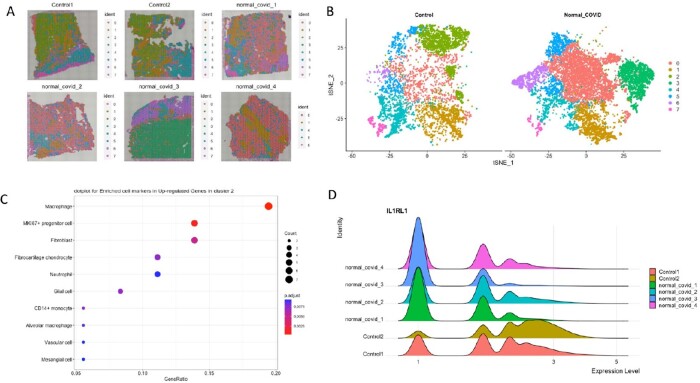
Enrichment of macrophages and interleukin 1 receptor-like 1 gene in unique cluster present in control placentas. A) Clusters overlying placental slides are shown. B) Control placentas with normal histopathology (n=2) on left and SARS-CoV-2 (COVID) placentas with normal histopathology on right ((n=4). C) Plot of enriched cell marker genes upregulated in cluster 2 highlights macrophages, MKi67+ progenitor cells, and fibroblasts. D) IL1RL1 gene is shown as an example of the set of genes significantly upregulated in control placentas compared to SARS-CoV-2 placentas.

**Figure 3.**
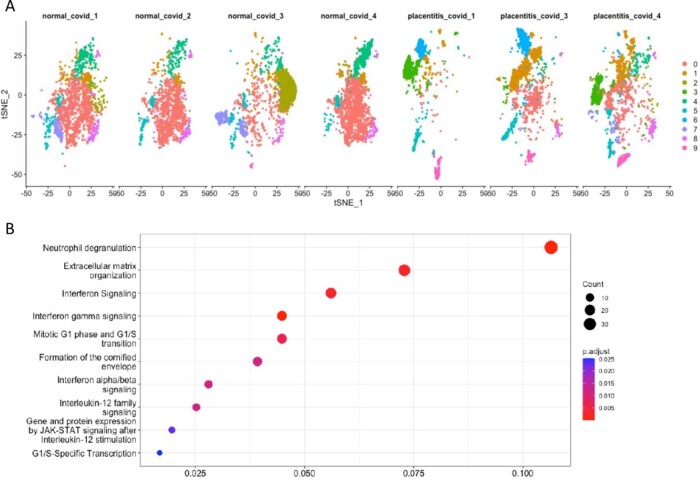
SARS-CoV-2 placentitis cases demonstrate increased expression of acute inflammatory activation compared to SARS-CoV-2 infection cases with normal histopathology. A) Gene expression cluster differences in normal SARS-CoV-2 (COVID) placentas and SARS-CoV-2 placentitis cases are shown. Placentitis shows decreased trophoblast transcripts and increased immune populations. B) Differential gene expression, especially surrounding live villi, demonstrate increased expression of acute inflammatory/immune activation genes including pathways such as neutrophil degranulation and multiple interferon families.

**Conclusion:**

This spatial transcriptomic analysis shows differential gene expression in control and SARS-CoV-2 placentas. SARS-CoV-2 placentitis demonstrates distinct acute inflammatory activation, providing pathophysiologic insight. Further pathway analysis of SARS-CoV-2-associated placental phenotypes is ongoing.

**Disclosures:**

**All Authors**: No reported disclosures

